# 454 antibody sequencing - error characterization and correction

**DOI:** 10.1186/1756-0500-4-404

**Published:** 2011-10-12

**Authors:** Ponraj Prabakaran, Emily Streaker, Weizao Chen, Dimiter S Dimitrov

**Affiliations:** 1 Protein Interactions Group, Center for Cancer Research Nanobiology Program, National Cancer Institute (NCI)-Frederick, National Institutes of Health (NIH), Frederick, MD 21702-1201, USA; 2 Basic Research Program, Science Applications International Corporation-Frederick, Inc., NCI-Frederick, Frederick, MD 21702, USA

## Abstract

**Background:**

454 sequencing is currently the method of choice for sequencing of antibody repertoires and libraries containing large numbers (10^6 ^to 10^12^) of different molecules with similar frameworks and variable regions which poses significant challenges for identifying sequencing errors. Identification and correction of sequencing errors in such mixtures is especially important for the exploration of complex maturation pathways and identification of putative germline predecessors of highly somatically mutated antibodies. To quantify and correct errors incorporated in 454 antibody sequencing, we sequenced six antibodies at different known concentrations twice over and compared them with the corresponding known sequences as determined by standard Sanger sequencing.

**Results:**

We found that 454 antibody sequencing could lead to approximately 20% incorrect reads due to insertions that were mostly found at shorter homopolymer regions of 2-3 nucleotide length, and less so by insertions, deletions and other variants at random sites. Correction of errors might reduce this population of erroneous reads down to 5-10%. However, there are a certain number of errors accounting for 4-8% of the total reads that could not be corrected unless several repeated sequencing is performed, although this may not be possible for large diverse libraries and repertoires including complete sets of antibodies (antibodyomes).

**Conclusions:**

The experimental test procedure carried out for assessing 454 antibody sequencing errors reveals high (up to 20%) incorrect reads; the errors can be reduced down to 5-10% but not less which suggests the use of caution to avoid false discovery of antibody variants and diversity.

## Background

The high-throughput 454 sequencing method has been applied for antibodies but errors associated with antibody repertoires and libraries are unknown and not yet quantified. Recently, antibodies from normal humans and patients have been sequenced and analyzed for identifying usage of different allelic genes, mutations and clonal expansions to help further our understanding of immune repertoire and clinical applications [1-3]. The extremely sensitive relationship between antibody sequence and function requires more accurate 454 sequencing. Antibodies have similar frameworks interspersed with highly variable regions and are formed by the recombination of two or three different genes, VJ for light and VDJ for heavy chains. Consequently, the available error correcting methods and algorithms developed for high-throughput sequence data [4-6] may not be relevant to 454 antibody sequencing. Although potential errors due to single nucleotide substitution and small InDels, insertion and deletion, associated with 454 pyrosequencing are known [7], exact error quantification and measurement of precision is not possible without conducting repeat sequencing runs of known antibody sequence target. Therefore, we performed 454 sequence analyses of six different antibodies at varied concentrations twice over and compared the reads with the original sequences determined by standard Sanger sequencing. This allowed us to identify the types of errors and estimate error rates, and suggest corrections applicable to 454 antibody sequencing for better confidence in the assessment of data quality.

## Results and Discussion

The antibodyome approach through 454 sequencing in exploring complex maturation pathways and identification of putative germline predecessors of highly somatically mutated antibodies could be useful in the development of effective vaccines against diseases such as AIDS and cancer [8]. Identification and correction of errors in 454 antibody sequencing is therefore of critical importance. To characterize and correct the 454 antibody sequencing errors, we performed the 454 sequencing of six clonally-related antibodies of known sequences at different concentrations twice over and calculated the error rates by comparing them to the results obtained from standard Sanger sequencing. The number of sequences produced was found to be correlated with the estimated number of molecules based on the content of DNA and concentration (Table 1). Sample antibody #1 has the highest occurrence of sequences and results of its duplicate sequencing were used to assess sequence quality from the two independent runs. The high-throughput 454 sequence data obtained for the six antibodies were compared with their known sequences using the pair-wise sequence comparison by the BLAST method. This helped us to identify accurate reads as well as erroneous reads where we observed different types of errors and their frequencies such as point mutation due to insertion, deletion or substitution, and errors involving two or more than two nucleotides (see additional file 1 for error characterization; types of errors and their frequencies as found in antibodies #1-6). These results provided an assessment of types and frequencies of errors observed in 454 antibody sequencing of six antibodies #1-6 which were 3-fold serially diluted.

**Table 1:** 454 sequencing of six different antibodies at different concentrations (3-fold dilution) produced different number of sequences (number in parentheses denotes the results from the second run)

Control antibodies	Number of molecules	Number of 454 sequences
1	100	1567 (1337)
2	33	352
3	11	159
4	4	39
5	1	8
6	0.4	5

**Figure 1:**
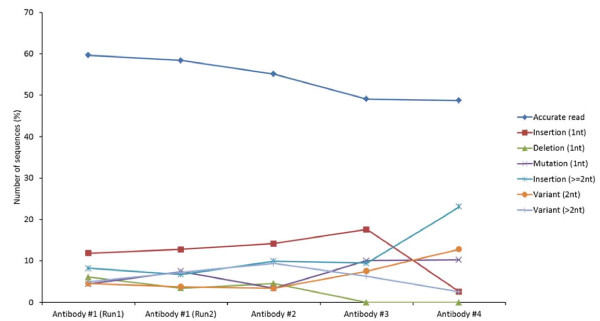
**The percentage values of accurate and erroneous reads with different types of errors observed in antibodies #1-4**. Antibody #1 was sequenced twice as two independent repeat runs, 1 and 2.

Our analysis revealed the different types of errors that could be incorporated during the 454 antibody sequencing. Antibodies #5 and #6 were not represented by a sufficient number of sequences to carry out a robust statistical analysis. Of the four antibodies #1-4, as depicted in Figure 1, we found that up to 60% of sequences as accurate reads and the remaining 40% had different types of sequence errors involving point mutations - insertion, deletion and substitution, and mixed error types involving more than 2 nucleotide changes. These data suggested that the amount of DNA used for the 454 sequencing did not significantly affect the observed errors. We further analyzed in detail the results from the two repeat runs of antibody #1 for more reliable statistics in computing the frequencies of different types of errors among erroneous reads. The single base-pair insertion was found to be the major cause of errors in ~12% of the total sequence reads from each data set which further increased to ~20% in combination with errors due to 2 or more insertions. Of the other types of point mutations, deletion and single substitution together accounted for 10% of errors while the errors due to variations with more than 2 nucleotides accounted for the remaining ~10%.

**Figure 2:**
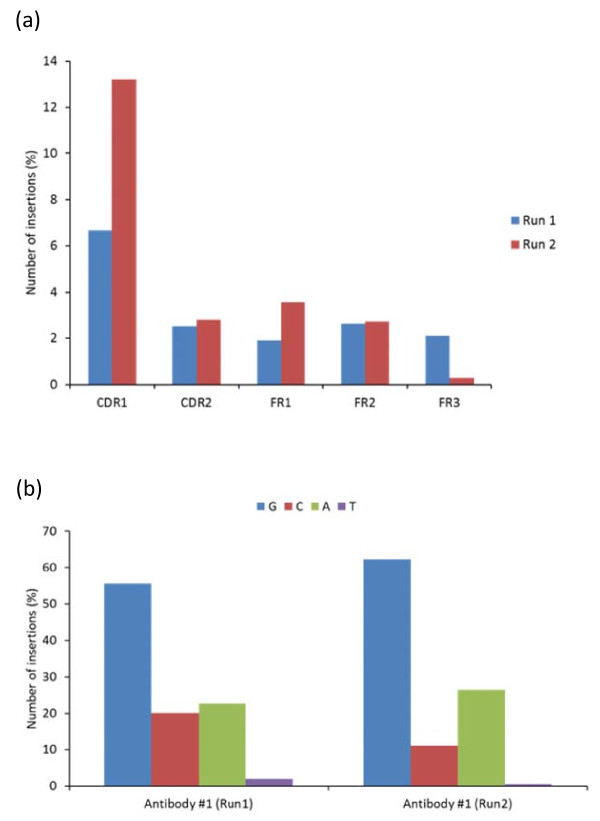
**(a), (b) Distribution of insertion errors and involvement of different types nucleotides, G, C, A and T, observed at the V-regions of antibody sequences from the two repeat 454 sequencing runs of antibody #1, which were detected and corrected by IMGT/HighV-QUEST analysis tool**.

To examine the consequence of these sequence errors, we performed IMGT/HighV-QUEST analysis of sequences for Run 1 and 2, and the results were summarized in Table 2. Most of the errors caused frameshifts, stop codons and unproductive rearrangements, resulting in functionally unproductive antibodies in more than 30% of the total reads. Also, we could identify, locate and correct errors in the V-region introduced by insertions and deletions using the IMGT/HighV-QUEST analysis tool. This error correction process using the IMGT tool could able to recover 16% and 23% of the sequence data from Run 1 and 2, respectively. The distribution of insertion errors along the V regions, frameworks (FR1-3) and complementarity determining regions (CDR1 and 2), of the antibodies indicated that sequence data from Run 2 had almost double the insertion errors at the CDR1 than from Run 1 (Figure 2a). This type of asymmetric incorporation of errors appeared in the repeated sequencing that was previously reported for another sequencing platform [6]. Further, to determine whether any specific types of errors observed were common among the two runs of antibody #1 as well as in others, we analyzed the specific locations and types of nucleotides involved in the insertion and deletion errors. We found that the nucleotide G, C and A were consistently inserted causing sequencing errors as shown for the runs 1 and 2 of antibody #1 (Figure 2b). We observed more than 50% of insertion with G and mainly occur at the site of polyG residues. Specifically, we found the most common errors which occurred due to insertions at either codon positions 27 and 29 of CDR1 regions through insertion of "G" and "A", respectively, in both the 454 data sets of runs1 and 2 for antibody #1. These two specific insertion errors occurred at polyG hotspots accounted for 30% in the run 1 and 50% in the run 2. We noted similar observation of errors but occurred at different sites of polyG as well as polyC, polyA and polyT in multiple reads, as shown for an example from the run #1 of antibody #1 (Table 3). This indicated that most of the insertions and deletions could even occur at relatively shorter homopolymer regions of 2-4 nucleotide length. Further, these errors were found to be distributed across the V-D-J regions (See additional file 2). We noted several errors due to insertion and deletion at the homopolymeric regions at the other antibodies #2-6 also (data not shown). Generally, these types of positional dependent insertion or deletion errors at homopolymeric regions in the antibody sequences can be detected and corrected as most of the errors led to frameshifts modifying the conserved cysteines and tryptophan residues. However, we observed that only 8% of sequences in run 1 and 2% in run 2 of antibody #1 did not cause frameshifting and led to altered nucleotide sequences. But, those altered reads could be still identified by IMGT tool which compares the user sequences with germline V genes to find the presence of insertion or deletion. Only errors in the CDR3 regions that do not cause any frameshifting and lacks significant identities to the D genes would be challenging for 454 antibody sequencing.

**Table 2:** The consequence of 454 antibody sequencing errors affecting the functionality of antibodies as determined by IMGT/HighV-QUEST using sequence data obtained from the two repeat 454 runs, 1 and 2.

Productive	65.8 (16.1)a	68 (23)a
Unproductive	13.7	5.3
Stop codon	3.7	3.1
No rearrangement	0.6	0.5
Functionality	Run 1 (%)	Run 2 (%)
Productive	65.8 (16.1)a	68 (23)a

^a^The percentage value of additional productive sequences recovered from the pool of unproductive sequences by correcting the errors due to mainly insertions.

**Table 3:** Insertion/deletion occurred in multiple reads mostly at homopolymeric regions of 2-3 nucleotide length as well as at the random sites, for example, shown for run 1 of control antibody #1.

Number of Sequences	Homopolymeric region?	Description of variation
6 sequences	yes: 3G in place of 4	1 deletion
7 sequences	yes: 4A in place of 3	1 insertion
8 sequences	yes: 4T in place of 3	1 insertion
9 sequences	yes: 4G in place of 2; no: 1 random G	2 + 1 insertion
11 sequences	yes: 3G in place of 4	1 deletion
21 sequences	yes: 4G in place of 3	1 insertion
22 sequences	yes: 4G in place of 3	1 insertion
28 sequences	no	1 deletion
31 sequences	yes: 4C in place of 3	1 insertion
32 sequences	yes: 3G in place of 2; no: 1 random G	1 + 1 insertion
42 sequences	yes: 3G in place of 2	1 insertion
49 sequences	no	1 deletion

**Figure 3:**
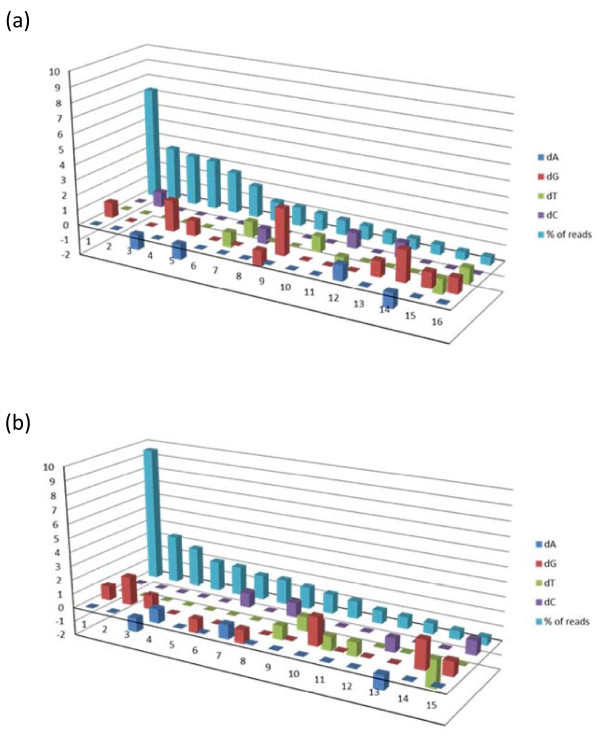
**(a), (b) The nucleotide composition analysis showing compositional differences in A, G, T and C (dA, dG, dT and dC) resulting from insertion, deletion or substitution or by combination as observed in erroneous sequence reads, respectively, from Run 1 and 2 of antibody #1**. Compositional differences are denoted by numeric values with "+" sign for overrepresentation and "-" sign for underrepresentation at the vertical axis and the total number of recurring erroneous reads in percent at the horizontal axis.

We performed nucleotide composition analysis of 454 sequences to find out whether any significant preference among nucleotides does exist for the observed point mutations. We noted some preferences for certain type of nucleotides either alone or in combination with others found inserted, deleted or substituted recurrently in more than 0.5% of erroneous sequences from Run 1 and 2 (Figure 3). For example, we noted a single nucleotide "G" was inserted (dA = 0, dG = 1, dT = 0 and dC = 0, shown at position 1 of the horizontal axis) in 7% and 10% of the sequences from Run 1 and 2, respectively, which was found to be appeared at the conserved codon position 27 as found by the IMGT/HighV-QUEST. The other recurring erroneous sequences ranged from 0.5% to 3% which also had specific usage of particular nucleotides.

Importantly, we found that the errors caused by single nucleotide substitution were difficult to identify and there could be a limitation for the error corrections. To locate these errors, we performed multiple sequence alignment of erroneous sequences with single substitution errors, which indicated that these errors distributed stochastically along the frameworks and CDR regions, up to 4% and 8% for Run 1 and 2, respectively. The distribution of these substitution errors are shown in additional file 3. Most of these single substitutions resulted in replacement mutations and therefore could not be easily detected unless they cause changes to the invariant residues such as cysteine and tryptophan which define the boundaries of the CDR3 as observed in a few cases. Similarly, mixed variants were identified to have 2 or more changes involving different error types at a rate of ~10% which, with the exception of insertions or deletions, would have been difficult to detect and rectify by post-computational processing.

## Conclusions

Our findings indicate that 454 antibody sequencing produces about 60% accurate reads which could routinely be improved above 80% by correcting insertions/deletions occurring at homopolymer sites as well as random sites, if represented by multiple reads and resulting in frameshifts, stop codons or modification of conserved residues. We noted that other types of errors caused by errors involving 2 or more nucleotide changes might be challenging; however, the use of post-processing methods such as IMGT V-QUEST and other antibody-specific algorithms to detect and rectify the errors to be developed might improve the accuracy up to 90-95%. The randomly occurring single nucleotide substitution errors accounting for 4-8% of the total reads as observed in run 1 and 2 of the larger data sets of control antibodies caused replacement mutations that in many instances can not be detected. These replacement errors may contribute to the anticipated residual errors after the post-sequencing error correction. Therefore, this type of errors could potentially lead to false discovery of novel variants or diversity unless verified with repeated runs or observed in multiple clonally-related sequences. We suggest that only those single nucleotide substitutions affecting the invariant residues of the highly-conserved frameworks as well as those residues at the complementarity determining regions that are conserved among the alleles and clonally related sequences among polyclonal repertoires may be detected and corrected. These results could be useful for identification and correction of 454 antibody sequencing errors.

## Methods

We used primers that were synthesized to include the Roche A and B adaptor sequences along with target amplification sequences: ControlF- 5'-CCATCTCATCCCTGCGTGTCTCCGACTCAGGCCACCAGCCATGGCC-3' (sense primer) and HR2:5'-/5BioTEG/- CCTATCCCCTGTGTGCCTTGGCAGTCTCAGGTCACAAGATTTGGGCTCAAC -3' (antisense primer) where 5BioTEG is a 5'-biotin-TEG moiety conjugated to the 5' end of the primer. The gene fragments were PCR amplified through 12 cycles and other details were followed according to the Roche 454 sequencing technical bulletin. Six different DNA samples encoding for antibody heavy chains of different lengths of known sequences (see additional file 4 for sequences in FASTA format) were prepared at 3-fold serial dilution and subjected to pyrosequencing using the Roche/454 Genome Sequencer FLX. The numbers of molecules in antibody #1 were empirically estimated based on the content of DNA and concentration which was approximately set to be 100. The numbers of molecules present in the remaining antibodies were calculated by taking into account of the 3-fold serial dilution. The 454 sequence data were trimmed for quality and only full-length sequences covering the entire antibody variable domain, F_V _region consisting all three complementarity determining regions (CDRs) along with frameworks (FRs), were retained (see additional files 5 and 6 for sequences in FASTA format). Sequence identities were calculated based on the pairwise alignment for each of 454 antibody sequence against pertinent known DNA sequences using local BLAST implemented in BioEdit v7.0.9 [9] with additional parameters, -G-1 -q-1 -r2. The BLAST output data comprising the start and end points of query and subject, and the number of mismatches including or excluding gaps were used to determine the different types of errors introduced during 454 pyrosequencing of antibodies. The IMGT/HighV-QUEST analysis tool [10] was extensively used to analyze 454 antibody sequence data for (i) locating the insertion and deletion errors along the antibody variable domain, (ii) identifying the consequences of such errors including the number of productive and unproductive genes, stop codons and no rearrangements, and (iii) correcting the antibody sequence errors which can be achieved by selecting the option "search for insertions and/or deletions" of advanced parameters available in the tool. The output results were stored in CSV files containing functionality information, list of insertion and deletion errors, and DNA as well as translated amino acid sequences after applying the appropriate sequence corrections. The algorithm and method implementation in IMGT/HighV-QUEST tool for determining the antibody functionality and correcting the antibody sequence errors were described previously [11]. Briefly, the frequencies of productive and unproductive sequences are calculated based on the absence of stop codons with in-frame junctions and stop codons with or without out-of-frame junctions, respectively. For correcting the antibody sequence errors, the IMGT tool uses two alignment steps (Smith and Waterman algorithm [12]) comparing the user sequence and the closest V genes and alleles in the database. This would reveal the frameshifts caused by sequencing errors to be fixed. First, if insertions are detected, they are excluded from the user sequence as they are not compatible with IMGT numbering and their locations are identified. If deletions are detected, gaps are introduced in the user sequence to restore the IMGT numbering. After insertion and/or deletion detection steps, the identification of V gene and allele are performed again and corrected sequences are provided. Also, we computed the nucleotide compositions for all reads from two sequencing runs of antibody #1. Statistical calculations were carried out using SAS JMP9^® ^statistical software (SAS Institute, Cary, NC) as well as Excel macros using the results as obtained from the BLAST and IMGT/HighV-QUEST. Graphical plots were made using Microsoft Excel 2010.

## Competing interests

The authors declare that they have no competing interests.

## Authors' contributions

DD conceived the study and WC constructed cDNA antibody gene fragments. PP and ES carried out computational analysis of 454 antibody sequence data. PP and DD wrote the manuscript. All authors read, corrected, and approved the final manuscript.

## Supplementary Material

Additional file 1 **454 antibody sequencing errors observed in six antibodies #1-6**. The percentage values of accurate reads and erroneous reads with which types of errors, such as insertion, deletion, substitution and variants with 2 and more nucleotide changes were calculated from 454 sequence data of the six antibodies. Click here for file

Additional file 2 **Multiple sequence alignment highlighting the location of insertion/deletion errors**. The location of insertion/deletion errors at homopolymeric regions of 2-3 nucleotide length as well as random sites of multiple reads from run 1 of control antibody #1 is shown by multiple sequence alignment of erroneous sequences along with the control antibody #1. Click here for file

Additional file 3 **Multiple sequence alignment highlighting the distribution of substitution errors**. The distribution of substitution errors resulting into replacement mutations (amino acids in single-letter codes) and stop codons (marked with X) observed from run 1 and 2 of control antibody 1, were shown in (a) and (b) respectively. Click here for file

Additional file 4 **FASTA file of the six antibodies #1-6**. The sequences of six clonally-related antibodies which are known from the standard Sanger sequencing are given in the FASTA file. Click here for file

Additional file 5 **FASTA file containing 454 sequence data of six antibodies #1-6 (sequences of antibody #1 correspond to run 1 of antibody #1)**. 454 sequencing data derived from the six control antibodies at different concentrations as mentioned in Table 1. Click here for file

Additional file 6 **FASTA file containing 454 sequence data of run 2 of antibody #1**. 454 sequencing data derived from the six control antibodies at different concentrations as mentioned in Table 1. Click here for file

## References

[B1] Glanville J Zhai W Berka J Telman D Huerta G Mehta GR Ni I Mei L Sundar PD Day GM Cox D Rajpal A Pons J Precise determination of the diversity of a combinatorial antibody library gives insight into the human immunoglobulin repertoire Proceedings of the National Academy of Sciences of the United States of America 2009 106 48 20216 20221 10.1073/pnas.0909775106 19875695 PMC2787155

[B2] Boyd SD Marshall EL Merker JD Maniar JM Zhang LN Sahaf B Jones CD Simen BB Hanczaruk B Nguyen KD Nadeau KC Egholm M Miklos DB Zehnder JL Fire AZ Measurement and Clinical Monitoring of Human Lymphocyte Clonality by Massively Parallel V-D-J Pyrosequencing Science Translational Medicine 2009 1 12 12ra23 10.1126/scitranslmed.3000540 20161664 PMC2819115

[B3] Boyd SD Gaëta BA Jackson KJ Fire AZ Marshall EL Merker JD Maniar JM Zhang LN Sahaf B Jones CD Simen BB Hanczaruk B Nguyen KD Nadeau KC Egholm M Miklos DB Zehnder JL Collins AM Individual Variation in the Germline Ig Gene Repertoire Inferred from Variable Region Gene Rearrangements Journal of Immunology 2010 184 12 6986 6992 10.4049/jimmunol.1000445 PMC4281569 20495067

[B4] Ilie L Fazayeli F Ilie S HiTEC: accurate error correction in high-throughput sequencing data Bioinformatics 2011 27 3 295 302 10.1093/bioinformatics/btq653 21115437

[B5] Lassmann T Hayashizaki Y Daub CO SAMStat: monitoring biases in next generation sequencing data Bioinformatics 2011 27 1 130 131 10.1093/bioinformatics/btq614 21088025 PMC3008642

[B6] Nguyen P Ma J Pei D Obert C Cheng C Geiger TL Identification of errors introduced during high throughput sequencing of the T cell receptor repertoire BMC Genomics 2011 12 106 10.1186/1471-2164-12-106 21310087 PMC3045962

[B7] Kircher M Kelso J High-throughput DNA sequencing - concepts and limitations Bioessays 2010 32 6 524 536 10.1002/bies.200900181 20486139

[B8] Dimitrov DS Therapeutic antibodies, vaccines and antibodyomes Mabs 2010 2 3 347 356 10.4161/mabs.2.3.11779 20400863 PMC2881260

[B9] Hall TA BioEdit: a user-friendly biological sequence alignment editor and analysis program for Windows 95/98/NT Nucl Acids Symp Ser 1999 41 95 98

[B10] Alamyar E Giudicelli V Duroux P Lefranc MP IMGT/HighV-QUEST: A High-Throughput System and Web Portal for the Analysis of Rearranged Nucleotide Sequences of Antigen Receptors - High-Throughput Version of IMGT/V-QUEST JOBIM 2010 Paper 60

[B11] Brochet X Lefranc MP Giudicelli V IMGT/V-QUEST: the highly customized and integrated system for IG and TR standardized V-J and V-D-J sequence analysis Nucleic Acids Res 2008 36 W503 508 10.1093/nar/gkn316 18503082 PMC2447746

[B12] Smith TF Waterman MS Identification of common molecular subsequences J Mol Biol 1981 147 195 197 10.1016/0022-2836(81)90087-5 7265238

