# Supplementary material for: 454 antibody sequencing - error characterization and correction
Source: BMC Res Notes. 2011 Oct 12;4:404. doi: 10.1186/1756-0500-4-404 (PMC3228814; doi:10.1186/1756-0500-4-404)
Supplement: Additional file 1 — 454 antibody sequencing errors observed in six antibodies #1-6. The percentage values of accurate reads and erroneous reads with which types of errors, such as insertion, deletion, substitution and variants with 2 and more nucleotide changes were calculated from 454 sequence data of the six antibodies. [file 1756-0500-4-404-S1.PDF]

454 antibody sequencing errors observed in control antibodies #1-6

Control	Accurate read	Insertion (1nt)	Deletion (1nt)	Mutation (1nt)	Insertion (2nt)	Insertion (>2nt)	Variant (2nt)	Variant (>2nt)
Antibody #1 (Run1)	59.67	11.87	6.13	4.47	5.36	2.87	4.59	5.04
Antibody #1 (Run2)	58.41	12.79	3.44	7.48	4.26	2.5	3.81	7.31
Antibody #2	55.11	14.2	4.55	3.41	7.39	2.56	3.41	9.37
Antibody #3	49.06	17.61	0	10.06	6.92	2.52	7.55	6.28
Antibody #4	48.72	2.56	0	10.26	10.26	12.82	12.82	2.56
Antibody #5	57.14	0	14.29	0	0	0	0	28.57
Antibody #6	40	0	0	60	0	0	0	0
