# Supplementary figures and images for: 454 antibody sequencing - error characterization and correction
Source: BMC Res Notes. 2011 Oct 12;4:404. doi: 10.1186/1756-0500-4-404 (PMC3228814; doi:10.1186/1756-0500-4-404)

## Multiple sequence alignment highlighting the location of insertion/deletion errors

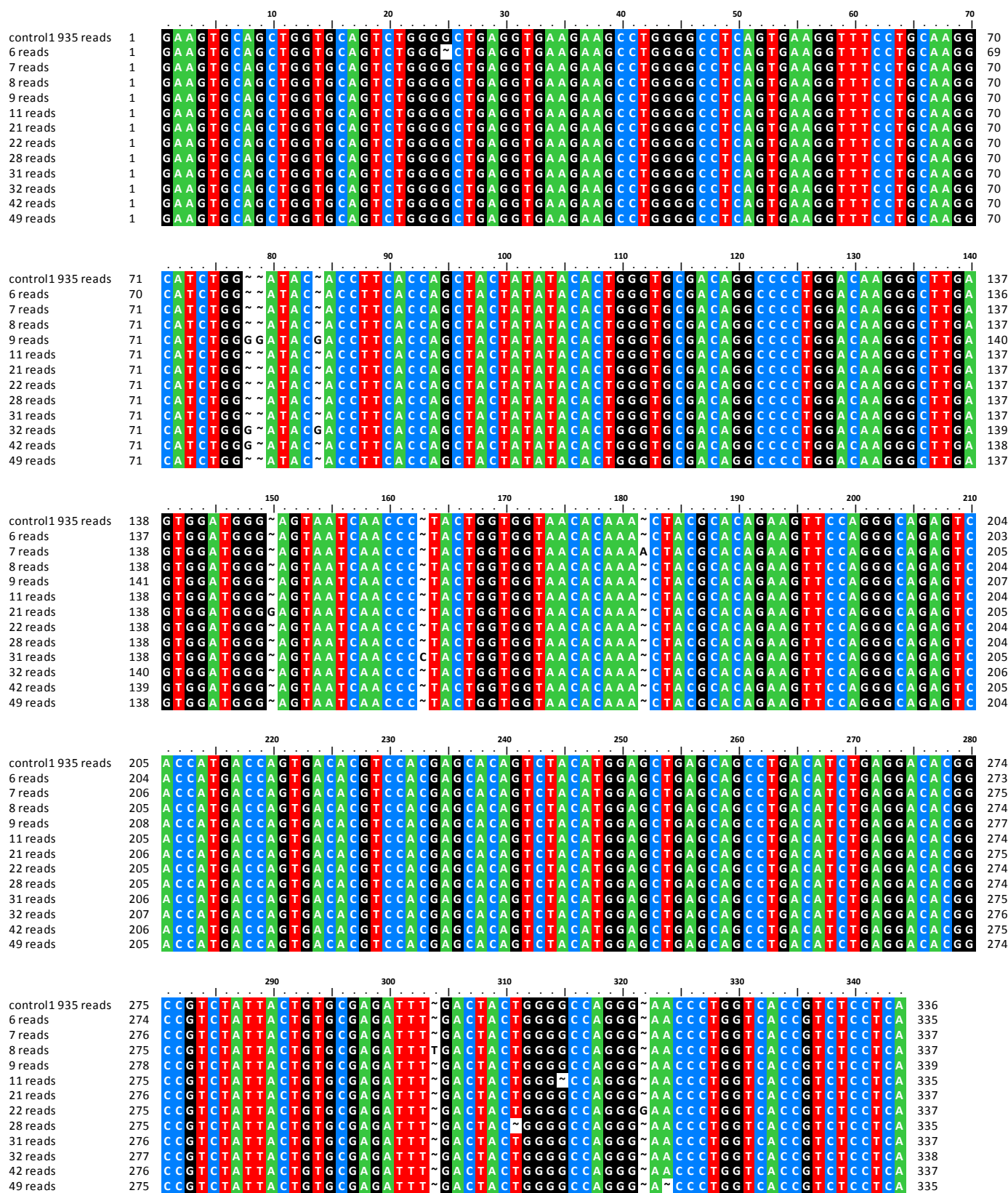

Supplement: Additional file 2 — Multiple sequence alignment highlighting the location of insertion/deletion errors. The location of insertion/deletion errors at homopolymeric regions of 2-3 nucleotide length as well as random sites of multiple reads from run 1 of control antibody #1 is shown by multiple sequence alignment of erroneous sequences along with the control antibody #1. [file 1756-0500-4-404-S2.PDF]

(a)

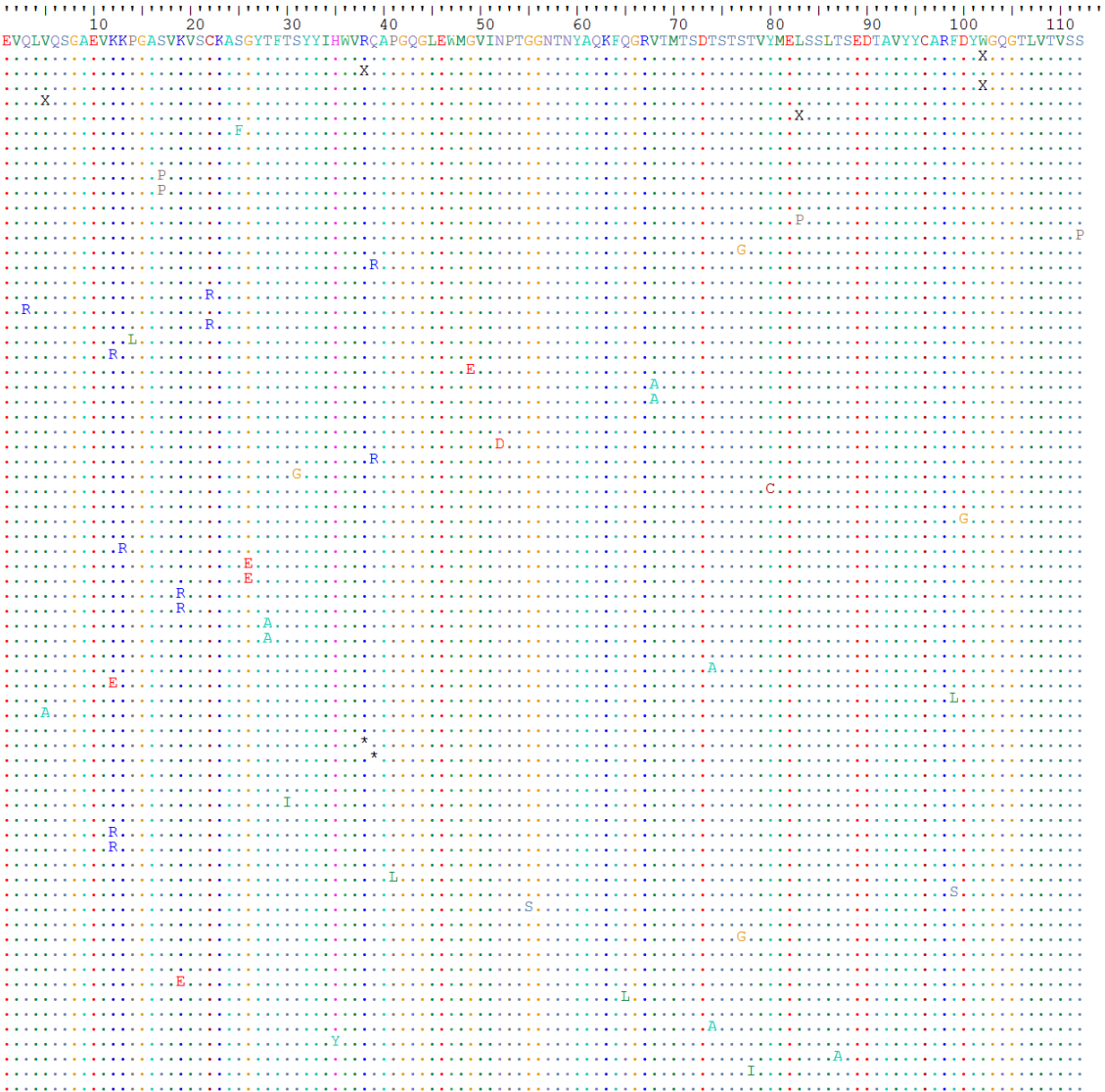

(b)

Supplement: Additional file 3 — Multiple sequence alignment highlighting the distribution of substitution errors. The distribution of substitution errors resulting into replacement mutations (amino acids in single-letter codes) and stop codons (marked with X) observed from run 1 and 2 of control antibody 1, were shown in (a) and (b) respectively. [file 1756-0500-4-404-S3.PDF]
